# Phytohormonal crosstalk: a plant defense network in response to abiotic and biotic stresses

**DOI:** 10.3389/fpls.2026.1990950

**Published:** 2026-10-30

**Authors:** Bushra A. Alhammad, Mahmoud F. Seleiman

**Affiliations:** 1 Biology Department, College of Science and Humanities, Prince Sattam Bin Abdulaziz University, Al Kharj, Riyadh, Saudi Arabia; 2 Department of Plant Production, College of Food and Agriculture Sciences, King Saud University, Riyadh, Saudi Arabia

**Keywords:** abiotic stress, biotic stress, hormonal regulation, phytohormones, plant defense, signaling

## Abstract

Plants are continuously subjected to a variety of environmental stresses, including biotic (such as herbivores and pathogens) and abiotic (such as drought, salinity, heat and cold stresses). Their ability to survive under such adverse conditions relies on highly coordinated and sophisticated phytohormonal signaling networks. Major plant hormones including abscisic acid (ABA), ethylene (ET), salicylic acid (SA), auxins, jasmonic acid (JA), gibberellins (GAs) and cytokinins (CKs) are involved in stress perception, defense mechanisms, growth, and development of plants. These hormones modulate gene expression, activate signal transduction cascades, regulate metabolic pathways and control key physiological processes such as reactive oxygen species (ROS) detoxification, osmoprotectant accumulation and stomatal movement. Evidence is accumulating that hormonal signaling pathways extensively cross-talk, enabling plants to balance growth and stress responses under unfavorable conditions. Moreover, phytohormone-mediated regulatory mechanisms are closely related with epigenetic modifications, RNA-based regulation and transcription factor networks that are involved in stress memory and priming, enabling plants to respond more quickly and efficiently to repeated stress events. This review summarizes the current knowledge of the roles of phytohormones in plant stress responses, especially their molecular mechanisms, signaling integration and hormonal interactions. Despite substantial progress, major knowledge gaps remain in understanding how interconnected hormonal networks integrate with transcriptional, epigenetic, metabolic, and redox processes, particularly under combined, fluctuating, and recurrent stresses. Future research integrating multi-omics, advanced phenotyping, systems biology, and precision breeding will be essential for resolving these context-dependent regulatory networks and translating mechanistic insights into high-yielding, climate-resilient crops.

## 1 Introduction

The rapid increase of the human population and the accelerating climate change have highlighted the pressing need to drastically increase crop productivity. The world population is forecast to be over 9 billion by 2050, with a 70% increase in food production necessary to satisfy the future demand (Francini and Sebastiani, 2019). The situation is made even more difficult by the frequency, the magnitude and the complexity of the environmental stresses affecting agricultural systems. Plants are subjected to several environmental constraints during their life cycle which have an adverse effect on growth, development and ultimately crop yield (Seleiman et al., 2021).

Salinity, waterlogging, drought, extreme temperatures, heavy metal toxicity and excessive solar radiation are the major abiotic stresses that severely affect plant growth and productivity and cause considerable reductions in biomass accumulation and grain yield under field conditions (Umar et al., 2021). Among these, salinity is especially harmful as it has negative effects on root growth and development, nutrient acquisition, and water uptake efficiency, leading to a decline in the overall productivity of plants. In addition to abiotic constraints, biotic interactions can have both positive and negative impacts on plant growth and productivity. Plants can benefit from associations like symbiosis with microorganisms, which can enhance performance and stress tolerance. Conversely, negative interactions with herbivores, pathogens and allelopathic organisms can severely restrict plant growth and reduce agricultural productivity (Natesan et al., 2023). During evolution plants have developed sophisticated surveillance and defense systems that enable them to sense and respond effectively to invading organisms. Systems of this kind depend on highly specific recognition mechanisms capable of detecting conserved molecular signatures of pathogens and herbivores. The conserved molecules include flagellin, peptide epitopes, glucans, glycoproteins and chitin derived fragments from fungal cell walls. These molecules together are called pathogen-associated molecular patterns (PAMPs) and are major elicitors of plant immune responses (Abdul Malik et al., 2020).

PAMP-triggered immunity (PTI), which is the first line of defense against invasive pathogens, is triggered when pattern recognition receptors (PRRs) on the surface of plant cells recognize PAMPs. Many pathogens secrete effector proteins to disrupt host immune signaling and facilitate infection in an effort to undermine PTI (DeFalco and Zipfel, 2021). Plants have in turn evolved a second layer of defense, called effector-triggered immunity (ETI), that allows them to recognize pathogen effectors and mount a strong, often localized, defense response (Abdul Malik et al., 2020). Damage-associated molecular patterns (DAMPs), which are endogenous chemicals generated from injured plant tissues, can trigger plant immune responses in addition to PAMPs and pathogen effectors. DAMPs are often released after pathogen attack or herbivore feeding, frequently through the action of enzymes degrading plant cell walls (Tanaka and Heil, 2021). These multilayered immune signaling pathways are intimately intertwined with the phytohormonal regulatory networks that govern plant growth, development, stress adaptation and defense responses. Phytohormones are thus central to balance productivity and immunity under changing environmental conditions (Kapoor et al., 2020).

The development and growth of an organism is determined by the intricate relationship that exists between genes and the environment. Plants have evolved complex processes to sense, react, and adapt to a variety of environmental issues since they are sessile organisms that cannot flee from unfavorable environmental conditions. Such adaptive responses allow plants to sense environmental changes quickly and respond by altering their physiological, biochemical and molecular processes (Chauhan and Singh, 2024). Central to these responses are complex signaling networks regulated by phytohormones and transcription factors that integrate stress perception and downstream defense mechanisms.

Plant growth regulators commonly known as phytohormones are key signaling molecules that control development, growth, and reactions to external stimuli at extremely low concentrations. These chemical messengers are essential for the productivity of plants and the tolerance against abiotic and biotic stresses (Ninkovic et al., 2021). Changes in biosynthesis, metabolism, transport, perception and signaling pathways of phytohormones can significantly impact plant performance under stress conditions. Therefore, phytohormones are known as the key regulators that integrate the development processes along with stress adaptation and thus contribute significantly to the plant survival and productivity under the changing environments.

Auxin, ABA, ET, GAs, SA, JAs, CKs, strigolactones (SLs) and brassinosteroids (BRs) are the main groups of plant hormones (Su et al., 2017). Auxin was the first phytohormone to be discovered (Su et al., 2017). Of these hormones, ABA, JA, ET and SA have been extensively studied in light of their central roles in mediating plant responses to environmental stresses. Plant adaptation to water deficit and other abiotic stresses is regulated via complex cellular and molecular processes controlled by phytohormones including ABA, SA, BRs, JAs, CKs, ET and GAs, which function via interconnected signaling networks (Hussain et al., 2023). The significance of hormones that were just recently discovered, including BRs and SLs, has received a lot of attention lately since they are crucial in assisting plants in adapting to adverse environmental conditions (Mao et al., 2023).

Therefore, the review provides an in-depth analysis of the roles of phytohormones in mediating plant responses to biotic and abiotic stresses. It addresses the molecular basis, signaling pathways and intricate crosstalk between major phytohormones that allow plants to perceive, integrate and respond to complex and often simultaneous environmental obstacles. This review aims to integrate recent developments in the field, to discuss critical knowledge gaps and emerging research directions and to provide a conceptual framework for future work. Furthermore, it provides useful information for the development of stress-resilient and high-yielding crop varieties needed for sustainable agricultural productivity under the mounting pressures of global climate change.

## 2 Phytohormonal regulation in plant stress tolerance

Plants respond to environmental stress by initiating a series of early signaling processes that, through the action of phytohormones, result in coordinated transcriptional, metabolic, physiological, and developmental responses. In addition to such primary intracellular stress signals like calcium fluxes, reactive oxygen species (ROS), protein phosphorylation cascades and dynamics of ion transport, phytohormones function as an integrative regulatory layer that determines the overall adaptive strategy of the plant (Devireddy et al., 2021). Hormonal regulation of plants plays an important role in water conservation, activation of defense mechanisms, cellular protection, growth inhibition, architectural modifications and post stress recovery. Therefore, phytohormone signaling is not just a downstream consequence of stress perception but a central regulatory node coordinating whole-plant adaptive responses under adverse environmental conditions.

Among the phytohormones, abscisic acid (ABA) is most closely associated with tolerance of abiotic stresses such as drought, salinity, osmotic stress and temperature extremes. ABA controls a wide range of adaptive responses such as osmotic adjustment, stomatal closure, membrane stability maintenance, hydraulic signaling, antioxidant defense and activation of stress-responsive gene expression (Samanta and Roychoudhury, 2024). One of the best-characterized pathways of ABA signaling involves the PYR/PYL/RCAR receptor family, protein phosphatase 2Cs (PP2Cs), and SNF1-related protein kinase 2 (SnRK2) kinases, which efficiently couple the perception of stress to downstream physiological and transcriptional responses (Fidler et al., 2022). Besides its canonical function as a stress hormone, ABA is a systems level regulator of root-to-shoot communication, redox homeostasis and growth suppression pathways (Zhang et al., 2020a). Under long-term stress, ABA-dependent survival responses often occur at the expense of biomass accumulation, photosynthetic capacity, and cell expansion, illustrating the inherent tradeoff between stress protection and productivity (Khan et al., 2025).

Salicylic acid (SA), jasmonates (JAs) and ethylene (ET) are other phytohormones that play important roles in plant adaptation to biotic and abiotic stresses (Pigolev et al., 2023). Jasmonates are mainly implicated in defense reactions against herbivorous insects and necrotrophic pathogens, besides adaptation to abiotic stresses and metabolic reprogramming (Roychowdhury et al., 2025). Ethylene regulates many physiological processes including defense responses, senescence, flooding tolerance and cell wall remodeling under stress conditions (Peerzada and Iqbal, 2021). Although these hormones are often classified according to certain biological functions, the regulatory roles of these hormones extend far beyond such simplistic classifications. Salicylic acid is involved in redox homeostasis and immune signaling, jasmonates regulate metabolic adjustment and long-term resource allocation, and ethylene is involved in the regulation of root architecture, tissue differentiation, and recovery from environmental stress (Kanwal et al., 2024) (**Figure 1**).

**Figure 1:**
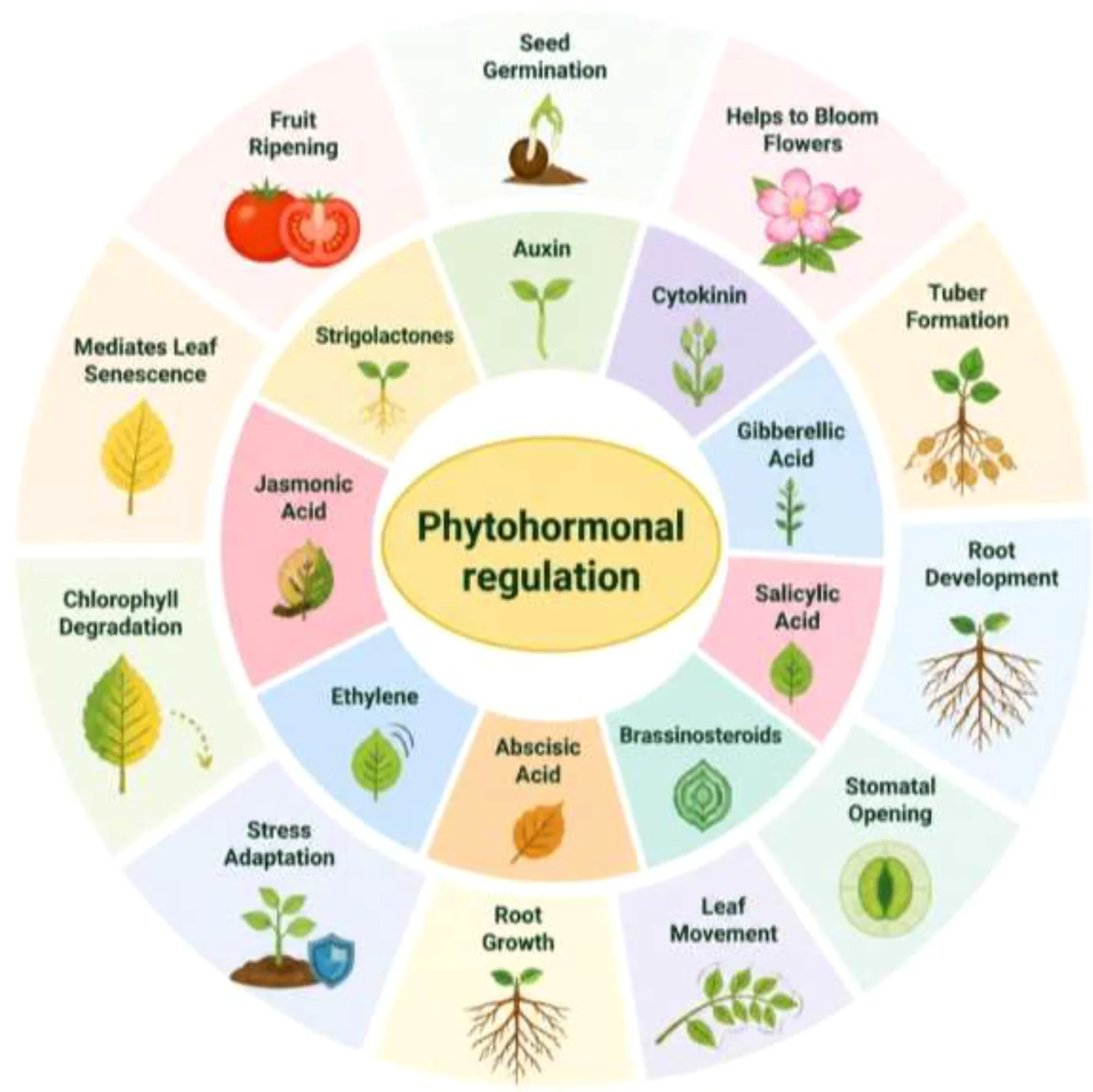
Describing the role of various phytohormones to regulate plants mechanisms.

Moreover, gasotransmitters regulate the phytohormone signaling, especially in the control of guard cell function and stomatal dynamics (Wang et al., 2022). L-cysteine desulfhydrase activity leads to the production of hydrogen sulfide (H_2_S), a key component of guard cell signaling networks that participates in ABA-dependent drought responses. In some cases, H_2_S has been proposed to act upstream of nitric oxide (NO) in stomatal closure. However, in Arabidopsis, the interactions of gasotransmitters are integrated into the ABA signaling pathway, where DES1-dependent H_2_S production is essential for ABA-induced NO accumulation and effective stomatal closure (Zhang et al., 2020b). In addition, H_2_S promotes ABA signaling by persulfidation of SnRK2.6/OST1 kinase, whereas NO might inhibit ABA responses through S-nitrosylation of the same target protein. These results indicate that H_2_S and NO may act in a series, antagonistically or cooperatively, depending on the particular molecular targets and physiological context (Wang et al., 2023). Moreover, NO-mediated S-nitrosylation has been associated with signaling pathways related to a number of phytohormones, such as SA, auxin, brassinosteroids, jasmonates, ethylene and cytokinins (Ergashev et al., 2025). Taken together, these observations support the notion that gasotransmitters are important modulators that fine-tune phytohormonal signaling networks.

Phytohormones are also strongly associated with plant productivity and are known as the key determinants of stress-induced developmental plasticity. Auxin is a key growth regulating hormone which is responsible for maintaining root architecture, organ patterning, tissue regeneration and cell expansion under optimal and stressful conditions. Environmental stresses frequently alter auxin biosynthesis, transport, distribution, and signaling, which in turn influence developmental programs and limit growth (Gomes and Scortecci, 2021). Under drought and salinity stress, auxin homeostasis is dynamically regulated, contributing to optimization of root system architecture, modulation of gravitropic responses, and enhancement of resource acquisition efficiency (Leftley et al., 2021).

Cytokinins are important regulators of the growth/stress adaptation balance. Reduced cytokinin signaling generally improves stress tolerance by restricting shoot growth and altering source–sink relationships. However, excessive suppression of cytokinin activity might promote senescence and be detrimental to recovery from stress. Therefore, optimal levels of cytokinin are needed to sustain the photosynthetic capacity, meristem activity, and recovery from stress. Thus, cytokinins are not only growth promoting hormones but also important regulators of the trade-off between stress tolerance and productivity.

Gibberellins (GAs) and brassinosteroids (BRs) are further examples of the tight link between developmental regulation and stress acclimation. Under stress conditions, GA signaling is often downregulated, resulting in the stabilization of DELLA proteins that inhibit elongation growth, while redirecting resources to survival and defense processes (Sarwar et al., 2023). However, complete inhibition of GA activity is usually not desirable as some GA signaling is necessary for recovery, reproduction and proper organ development. Similarly, brassinosteroids enhance stress tolerance through the promotion of membrane stability, antioxidant capacity and cellular defense mechanisms (Sadura and Janeczko, 2021). Together these findings demonstrate that growth-promoting phytohormones are not merely antagonistic to stress tolerance. Instead, they regulate the degree of growth reduction, reprogramming or maintenance depending on the type, intensity and duration of environmental stress, thus allowing plants to optimize both survival and productivity (**Table 1**).

**Table 1:** Role of phytohormones to regulate abiotic stresses.

Crop	Hormones	Stress	Mechanism controlled	Effect on plants	References
Rice (*Oryza sativa* L.)	ABA	Cadmium stress	ABA regulates transpiration and Cd uptake	Enhanced Cd tolerance via reduced Cd accumulation	(Hsu and Kao, 2003)
*Brassica juncea*	ABA	Cadmium exposure	Cd induces ABA- and drought-responsive gene expression indicating ABA involvement in stress signaling	Activation of stress-response pathways under Cd stress	(Fusco et al., 2005)
*Arabidopsis thaliana*	ABA, Light	Seed germination and seedling development	HY5 integrates light and ABA signaling via ABI5 regulation	Alters ABA sensitivity and early seedling development	(Chen et al., 2008)
*Arabidopsis thaliana*	ABA	Salt (NaCl) and osmotic stress during germination	ABA biosynthesis gene ABA2 regulates endogenous ABA production	ABA-deficient mutants show enhanced germination and osmotolerance	(González-Guzmán et al., 2002)
*Halobacillus trueperi* (RSK CAS9)	Indole-3-acetic acid (Choudhary et al.), EPS-associated signaling	Salinity stress (NaCl)	EPS production and IAA synthesis promote osmotic protection, biofilm formation, and stress adaptation	Improved salinity tolerance via microbial growth promotion	(Loganathan et al., 2026)
*Triticum durum* (Durum wheat)	Indole-3-acetic acid (Choudhary et al.)	NaCl	Exogenous IAA enhances photosynthesis, K uptake, osmotic adjustment, and ionic homeostasis	Improved growth, yield traits under salt stress	(Hmissi et al., 2025)
*Glycine max* (soybean)	Indole-3-acetic acid (Choudhary et al.)	NaCl	Acinetobacter pittii YNA40 produces IAA, enhancing plant growth and stress adaptation	Improved growth, chlorophyll content, antioxidant activity, and salt tolerance	(Kang et al., 2024)
Malus spp. (Apple: *M. domestica* ‘Fuji’, *M. baccata*, *M. zumi*)	Auxin/Indole-3-acetic acid signaling (Aux/IAA genes)	NaCl	Salt-induced expression of MdIAA genes enhances auxin signaling and transcriptional stress response	Improved salt tolerance in tolerant genotypes and transgenic calli overexpressing MdIAAs	(Li et al., 2022c)
*Arabidopsis thaliana*	Indole-3-acetic acid (Choudhary et al.)	NaCl	Salt-specific activation of auxin biosynthesis (NIT2-mediated IAA production) regulates ionic balance (Na^+^/K^+^ ratio)	Enhanced growth, survival, and salinity tolerance via increased IAA levels	(Cackett et al., 2022)
Rice (*Oryza sativa* L.)	Cytokinin	NaCl	RST31/ABCG18 regulates root-to-shoot cytokinin transport, affecting ROS accumulation and DNA damage	Reduced shoot cytokinin lowers ROS and enhances salt tolerance	(He et al., 2024)
Tomato (*Solanum lycopersicum*)	Cytokinin, ABA	Drought stress	Cytokinin-producing *Pseudomonas fluorescens* G20–18 enhances ABA accumulation, stomatal closure, antioxidant activity, and stress-responsive gene regulation	Improved drought tolerance, growth, antioxidant defense, and recovery capacity	(Mekureyaw et al., 2022)
Rice (*Oryza sativa* L.)	Ethylene-responsive signaling (AP2/ERF pathway)	Drought and submergence stress	OsEBP89 regulates ROS scavenging, proline accumulation, and stress-responsive gene	Knockout of OsEBP89 enhances drought	(Zhang et al., 2022)
Alfalfa (*Medicago sativa*)	Ethylene	NaCl	Ethylene signaling via MsETR2 regulates antioxidant activity, proline accumulation, chlorophyll maintenance, and ERF gene expression	Improved seed germination and seedling growth under salinity stress	(Wang et al., 2020)
*Zea mays* (Maize)	Salicylic acid (SA)	Drought, heat, and combined abiotic stress	Stress alters SA metabolism and phenylalanine–shikimate pathway affecting root growth and SA signaling	Reduced primary root growth and metabolic reprogramming under stress	(Yang et al., 2022)
*Trigonella foenum*-graecum (fenugreek)	SA	Heat stress (30–40 °C)	SA modulates antioxidant system, protein expression, membrane stability, photosynthesis, and stress proteins	Improved thermotolerance and mitigation of heat-induced damage	(Choudhary et al., 2024)
*Arabidopsis thaliana*	Jasmonic acid (JA)	Heat stress	HSFA2 regulates JA biosynthesis genes (LOX3, OPR3) via Ca²^+^ signaling cascade	Increased JA improves membrane stability, chlorophyll content, and survival under heat stress	(Guo et al., 2024)
*Grewia asiatica* L.	JA	Drought stress	JA enhances antioxidant enzyme system (APX, GPX, SOD), phenolic metabolism, and gas exchange regulation	Improved growth, CO_2_ assimilation, stomatal conductance, and drought tolerance	(Waheed et al., 2022)
*Glycine max* (soybean)	JA	NaCl	JA modulates phytohormone balance, ion homeostasis, antioxidant system, and photosynthesis	Improved physiological performance and salt tolerance	(Sheteiwy et al., 2021)
*Capsicum annuum* (chili)	SLs	NaCl	Under salt stress, strigolactone improves root growth, nutritional uptake (N, P, and K), and physiological control.	Improved growth, chlorophyll content, and yield under salinity	(Danish et al., 2024)
*Zea mays* (Maize)	SLs	Drought stress	Strigolactone improves gas exchange, water relations, photosynthetic efficiency, and antioxidant enzyme activity.	Improved drought tolerance via reduced oxidative damage and better water status	(Sattar et al., 2022)
Rice (*Oryza sativa* L.)	SLs-GR24	NaCl	Under salt stress, GR24 promotes water relations, photosynthesis, and antioxidant enzymes (SOD, POD).	Improved growth, chlorophyll content, and salt tolerance at optimal dose (1 µM)	(Ling et al., 2020)

### 2.1 Auxin-mediated regulation of stress adaptation in plants

Indole-3-acetic acid (IAA) is the predominant naturally occurring auxin in plants and is primarily synthesized in areas of active growth, such as shoot apical meristems, developing leaves, and young fruits (Khan et al., 2021). Recent studies have shown that root tips can also synthesize auxin locally, and together with auxin originating from the shoot, this is critical for regulating root architecture and developmental plasticity (Roychoudhry and Kepinski, 2022; Sarikhit et al., 2025).

Soil salinity is one of the major constraints to global agricultural productivity. Salt stress causes several forms of cellular damage such as osmotic stress, ion toxicity, oxidative stress, nutrient imbalance and metabolic disruption. Therefore, plants show growth retardation, low photosynthetic efficiency, high energy cost, premature senescence, and death in severe cases (Hameed et al., 2021; Nefissi Ouertani et al., 2022). Ion homeostasis and osmotic adjustment as well as the individual roles of hormones during salinity stress have been studied extensively, but less investigations have been done on hormonal crosstalk and auxin dynamics under saline conditions.

The effect of salinity on auxin biosynthesis, transport and accumulation is still complex and in some cases contradictory. Changes in auxin content under salt stress have been reported (Verma et al., 2022), but the direction and the extent of the changes seem to be species-, tissue type-, and stress intensity-dependent. Some studies have demonstrated that high IAA levels are associated with growth inhibition under salinity conditions (Emenecker and Strader, 2020), and others have observed significant reductions in IAA concentrations in wheat roots, tomato leaves and rice plants under salinity stress (Yemelyanov et al., 2020). By contrast, Wu et al. (2021) found that auxin levels in the apices of Arabidopsis roots and shoots were relatively stable under mild salt stress. Further evidence suggests that the Salt Overly Sensitive (SOS) signaling pathway is involved in the maintenance of auxin homeostasis and signaling stability under salinity stress (**Figure 2**).

**Figure 2:**
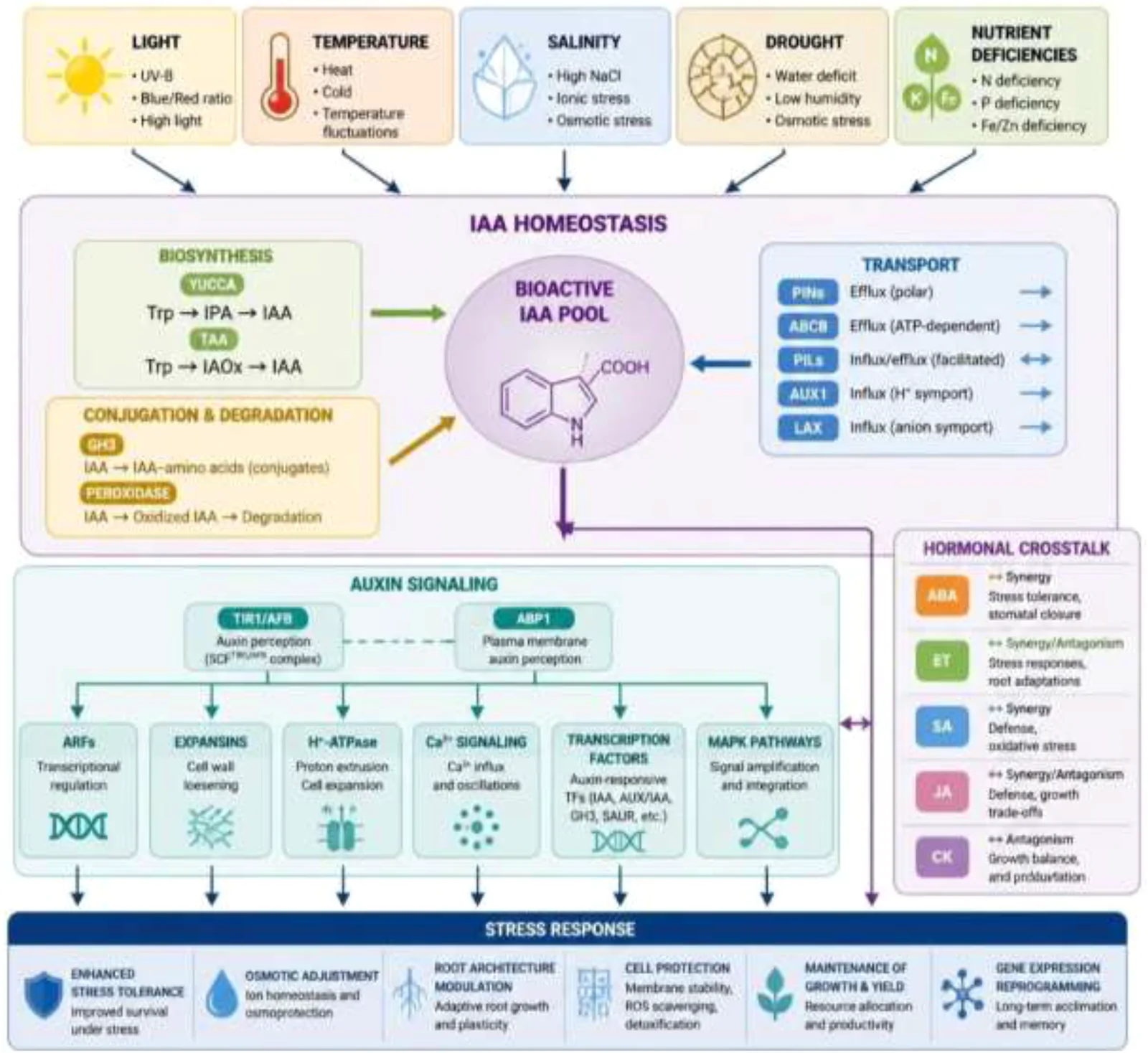
IAA synergy with various phytohormones homeostasis under different abiotic stress conditions and activating different signaling pathways.

There is increasing evidence that auxin is a major player in plant stress adaptation. Changes in auxin biosynthesis, transport, perception and signaling are important determinants of plant tolerance to adverse environmental conditions. A particularly important mechanism controlling plant responses to a wide range of abiotic stresses is the post-transcriptional regulation of AUXIN RESPONSE FACTOR (ARF) genes by small RNAs (Song et al., 2023b). In Arabidopsis, overexpression of the auxin biosynthetic gene YUC6 increases drought tolerance by increasing auxin levels, suggesting that high auxin levels and tryptophan-dependent auxin biosynthesis pathways are involved in drought adaptation (Gao et al., 2024).

Similarly, the silencing of the SlARF2 gene in tomato has been shown to increase tolerance to drought and salinity stresses. Downregulation of SlARF2 enhances root growth, improves plant water status, increases chlorophyll and soluble sugar contents, induces proline accumulation, alleviates oxidative damage, and activates stress responsive genes associated with root development. These findings suggest that SlARF2 functions as a repressor of abiotic stress tolerance and imply that modification of this gene could be a strategy to improve crop productivity under stressful environmental conditions (Yu et al., 2025).

PeFUS3 has been recently identified as a key regulator of drought adaptation in poplar. PeFUS3 regulates auxin and ABA signaling pathways and interacts with ABA-responsive transcription factors and induces the expression of auxin transport-related genes, thereby maintaining lateral root growth under water-deficit conditions. Thus, PeFUS3 is a possible candidate for enhancing drought tolerance in woody plant species (Liu et al., 2025). In a similar way, the auxin transporters ABCB28 and ABCB29 conduct auxin flux from chloroplasts to the cytosol, connecting auxin homeostasis to the regulation of photosynthesis, stomatal functioning and stress adaptation. Hence, these transporters could be novel targets for enhancing water-use efficiency and stress resilience in crops under drought and salinity stress (Tamizhselvan et al., 2023).

Plant development, responses to abiotic stress, and interactions between plants and microbes are all influenced by auxin. Although indole-3-acetic acid is a plant hormone, it can also act as a signaling molecule to change the behavior of microorganisms. Increased auxin levels may increase host susceptibility to some pathogens and increase pathogen virulence simultaneously. Thus, knowing how auxin signaling is controlled during infection could offer useful paths for increasing disease resistance in crops (Kunkel and Johnson, 2021).

Conversely, auxin signaling can also promote beneficial symbiotic interactions. Arabidopsis thaliana and the endophytic fungus Serendipita indica associate to promote plant growth by maintaining auxin signaling activity in roots. This process includes the strict regulation of auxin conjugation by GH3.5 and GH3.17 and the modulation of the PIN2 auxin transporter, ensuring optimal auxin homeostasis during symbiosis (González Ortega‐Villaizán et al., 2024).

### 2.2 ABA-mediated regulation of stress adaptation in plants

Abscisic acid (ABA) is a major regulator of plant growth, development and adaptation to environmental stress. It is involved in many physiological processes like seed dormancy, seed germination, stomatal regulation and plant productivity. Under unfavorable environmental conditions, ABA is known as “universal stress hormone” as it increases the production of protective metabolites and orchestrates various stress-responsive mechanisms (Singh et al., 2022).

ABA is a highly responsive stress phytohormone, which accumulates rapidly under osmotic stress conditions and plays a critical role in plant tolerance to drought, salinity, extreme temperatures, and other abiotic stresses. Besides its classical roles in abiotic stress signaling, ABA modulates several defense pathways involved in plant–microbe and plant–insect interactions. Moreover, ABA is a signaling molecule in interspecies communication in which it may suppress immune responses or promote beneficial associations such as arbuscular mycorrhizal symbiosis (Ganugi et al., 2019).

Some pathogens have evolved mechanisms to subvert ABA signaling to facilitate infection. For example, Pseudomonas syringae pv. Coronatine and type III effector proteins produced by the tomato pathogen DC3000 activate ABA-associated pathways but at the same time suppress mitogen-activated protein kinase (MAPK)-mediated immune responses and salicylic acid dependent defense mechanisms (Cao et al., 2011; Lim et al., 2014).

ABA acts as a master regulator of adaptive responses to drought stress. ABA accumulation leads to stomatal closure, thus decreasing transpirational water loss and increasing water-use efficiency. ABA is also involved in several developmental processes such as seed dormancy, root system architecture, besides its role in stress adaptation (Khan, 2025). The presence of delayed senescence phenotypes in several ABA-deficient mutants gives further evidence for the role of the hormone in the regulation of leaf aging and developmental transitions.

At the molecular scale, ABA accumulation induced by drought activates a large number of stress-responsive genes that help in osmotic adjustment, cellular protection and water conservation (Li et al., 2022a). Transcriptomic analyzes have shown a large overlap between drought-responsive and ABA-responsive gene networks. In Arabidopsis, about 245 genes are co-regulated by drought stress and ABA signaling. In rice, 43 of 73 drought responsive genes are also regulated by ABA. These results emphasize the critical importance of ABA in regulating transcriptional responses that allow plants to adapt to water-deficit conditions (Aslam et al., 2026).

### 2.3 Cytokinin-mediated regulation of stress adaptation in plants

Cytokinins (CKs) are multifunctional phytohormones that control numerous processes in plant growth and development. They are involved in seed germination, morphogenesis, chloroplast biogenesis, stomatal regulation, leaf and fruit abscission, and root-to-shoot communication. Cytokinins are not only involved in developmental processes but also play a role in the response of plants to environmental stresses. Alterations of the endogenous cytokinin levels under stress conditions indicate their involvement in adaptive processes that enable plants to maintain growth and productivity in adverse environments.

Increasing evidence indicates that the homeostasis of cytokinins is dynamically regulated under drought stress. In the early stages of drought, levels of cytokinin in xylem sap typically decrease, indicating a shift from growth-related processes to stress adaptation. However, responses are variable between cytokinin forms and plant species. For example, zeatin levels often do not significantly change until the second exposure to drought, and drought-sensitive tomato cultivars have a greater reduction in cytokinin content compared to tolerant genotypes (Septia et al., 2025). Onion and French bean also exhibit similar decrease in the levels of cytokinin. The extent of decrease is dependent on cultivar, development stage and severity of stress (Sansan et al., 2024).

Cytokinins biosynthesis is mainly regulated by isopentenyl transferase (IPT) which is a key enzyme responsible for the rate-limiting step of cytokinin synthesis. The increased expression of IPT results in increased cytokinin accumulation and has been associated with increased tolerance of drought and other environmental stresses. Moreover, approaches based on seed priming with cytokinin have shown potential to improve plant performance under salinity stress through enhancing physiological resilience and maintaining cellular homeostasis (Yadav et al., 2024).

Cytokinins are involved in salinity tolerance by several processes like regulation of senescence, maintenance of photosynthetic activity, regulation of root and shoot growth and interaction with other hormonal pathways especially ABA and ethylene signaling. Such interactions enable cytokinins to mediate the balance between growth and defense responses and enable plants to sustain productivity under saline conditions. However, responses of cytokinin to salinity are often species-specific suggesting the need to study the hormonal crosstalk and regulatory networks in salt-tolerant crops.

In recent years, several cytokinin-related regulators involved in adaptation to abiotic stresses were identified in molecular studies. OsPHP1, a rice gene as a negative regulator of cytokinin signaling and stress tolerance. Knockdown of OsPHP1 increases the antioxidant capacity, photosynthetic efficiency, growth and yield related traits under stress conditions, while overexpression leads to increase in stress sensitivity (Yadav et al., 2024). Likewise, CRF2 has been demonstrated to be important for salinity tolerance through the regulation of cytokinin biosynthesis, ion homeostasis and transcriptional reprogramming. CRF2 deficiency is detrimental to chlorophyll accumulation, photosystem II activity, and photosynthetic performance under salt stress, demonstrating the importance of CRF2 in hormonal signaling and physiological adaptation (Keshishian et al., 2022).

Additionally, it has been noted that exogenous cytokinin treatment holds significant promise for enhancing crop stress tolerance. Application of synthetic cytokinin 6-benzyladenine (6-BAP) enhances growth and productivity of drought-stressed soybean with stimulation of adaptive physiological responses and maintenance of photosynthetic performance (Mangena, 2022). These findings indicate that cytokinin-based treatments could provide practical solutions to mitigate abiotic stress and improve crop robustness.

Cytokinin oxidase/dehydrogenase (CKX), an enzyme that degrades cytokinin, also plays a role in the regulation of cytokinin homeostasis under stress. Regulation of CKX activity is essential for the balance between growth and adaptation to stress. High cytokinin degradation can impede plant growth and controlled cytokinin turnover is important for cellular homeostasis, defense-related metabolism and drought tolerance (Khablak et al., 2024). Altogether these results demonstrate the significance of cytokinins as integrators of developmental and environmental stress responses to allow plants to maximize growth and to cope with adverse conditions.

### 2.4 Role of ethylene in plant stress adaptation

Seed germination, root growth, gravitropism, flowering, fruit ripening, abscission, and senescence are just a few of the many physiological and developmental processes that are regulated by the simple gaseous phytohormone ethylene (ET). Ethylene biosynthesis is highly responsive to environmental cues and is commonly induced by abiotic and biotic stresses including wounding, flooding, hypoxia, salinity, drought and pathogen attack (Wang et al., 2025). Ethylene is a central stress-responsive hormone that regulates plant adaptation to adverse environmental conditions.

Ethylene accumulation under stress conditions is generally considered as an adaptive response as it allows the reprogramming of growth, the regulation of senescence and the allocation of resources for survival. However, effects of ethylene are highly context-dependent and vary with stress intensity, duration, developmental stage and plant species. Under normal conditions, moderate ethylene production enhances stress tolerance, but under severe or prolonged stress, ethylene production may be inhibited, or signaling pathways may be disrupted, thus compromising the stress response (Shekhawat et al., 2022).

Recent studies have indicated that ethylene is important for drought and salinity tolerance. Disruption of ethylene biosynthetic genes PhACO1 and PhACO3 in petunia leads to a dramatic reduction in ethylene biosynthesis and an increased sensitivity to drought and salt stress. These mutants show defects in physiological and molecular responses, pointing out the essential function of ethylene in integrating stress adaptation mechanisms (Al Azzawi et al., 2025; Tripathi et al., 2024). These results highlight the need for careful manipulation of ethylene signaling pathways in crop improvement programs since excessive suppression or enhancement of ethylene responses may have undesirable developmental consequences.

Ethylene also plays an important role in salinity tolerance by maintaining ion homeostasis. This contrasts with earlier concepts that focused on sodium exclusion. New evidence suggests that ethylene induces salt tolerance by increasing retention of potassium in roots and shoots, thus maintaining ionic balance at the cellular level. In addition, ethylene signaling is intimately linked with the Salt Overly Sensitive (SOS) pathway and other stress-responsive networks, implying its significant role in plant salinity adaptation. However, the molecular mechanisms linking ethylene signaling to ion transport and stress acclimation are not fully understood (Zhang et al., 2024b).

Ethylene regulates not only ion homeostasis but also the maintenance of cellular redox balance during stress. Ethylene signaling in tomato fruit regulates antioxidant enzymes activities and polyamine metabolism under salinity to alleviate oxidative damage and improve stress tolerance. Increased susceptibility is observed in ethylene receptor mutants such as the Never-ripe (Nr) mutant, further reinforcing the essential function of ethylene perception in stress adaptation (Takacs et al., 2022).

Ethylene Response Factors (ERFs), which control the expression of many stress-responsive genes, play a major role in mediating ethylene signaling at the transcriptional level. For instance, ERF34 increases the expression of stress-related genes involved in cellular protection, antioxidant defense, and delayed senescence to provide salt tolerance in Arabidopsis. This transcription factor plays a key role in connecting ethylene signaling with stress-responsive developmental programs (Park et al., 2022).

Ethylene has also been implicated in plant responses to low temperature stress; Increased ethylene production is often correlated with an increased cold tolerance via induction of protective metabolic pathways and stress-responsive gene networks. However, the precise molecular mechanisms of stress-induced ethylene biosynthesis and cross-talk with other phytohormonal signaling pathways are still under investigation. Improved knowledge of these regulatory networks will allow the development of crops that are more resilient to many environmental stresses.

### 2.5 Gibberellins as modulators of abiotic stress tolerance in plants

Ethylene Response Factors (ERFs), which control the expression of many stress-responsive genes, play a major role in mediating ethylene signaling at the transcriptional level. More than 130 gibberellins have been reported; among them GA_1_, GA_3_, GA_4_ and GA_7_ are considered to be the principal bioactive forms regulating the physiological processes in plants (Rahman et al., 2026). Gibberellins, in addition to their well-known functions in development, are also involved in plant responses to biotic and abiotic stresses.

The role of GAs in stress adaptation is indicative of their ability to integrate growth and survival under adverse environmental conditions. Gibberellin signaling regulates leaf area development, photosynthetic efficiency, nutrient utilization, and biomass accumulation, thereby linking environmental adaptation with crop productivity. Modulation of GA metabolism and signaling enables plants to balance growth demands with stress defense mechanisms under drought and salinity stress (Shahzad et al., 2021).

DELLA proteins are central repressors of gibberellin signaling and a crucial component of GA-mediated regulation of stress. Bioactive GAs promote growth-related processes under favorable growth conditions by stimulating DELLA degradation. Conversely, environmental stresses generally decrease GA levels, resulting in DELLA accumulation and growth inhibition. This switch allows plants to redistribute the metabolic resources from growth to stress tolerance and survival. Therefore, DELLA proteins are more and more considered as key regulators of the growth-defense trade-off during environmental stress.

Gene studies also confirm the role of GA signaling components for stress adaptation. The SPINDLY (SPY) protein acts as a negative regulator of abiotic stress tolerance in Arabidopsis. Loss-of-function spy mutants are more tolerant to drought and salinity, while SPY overexpression results in stress hypersensitivity. These antagonistic phenotypes are associated with changes in gibberellin signaling, cytokinin-responsive pathways and stress gene expression, and imply that SPY functions as a key integrator of hormonal crosstalk during adaptation to the environment (Qin et al., 2011).

Exogenous application of gibberellic acid (GA 3) has also been found to affect plant responses to environmental stress. In mild saline conditions, GA_3_ decreases the stomatal resistance and increases the water uptake of tomato plants, which in turn improves the physiological performance and tolerance to stress. Transcriptomic studies in Arabidopsis also revealed that GA signaling regulates many genes involved in osmotic stress adaptation and cellular protection (Tripathi et al., 2025).

However, the beneficial effects of GA are strongly concentration-dependent. Low concentrations of GA_3_ (3–5 mg L^−^¹) in spinach alleviate copper toxicity through enhanced antioxidant defenses, photosynthetic efficiency, biomass production, and copper sequestration in roots rather than shoots. However, at higher concentrations, they increase oxidative stress and tissue damage (Javed et al., 2021). Similarly, exogenous application of GA 3 (100 mg L -1) enhanced the growth, photosynthesis performance and water-use efficiency for both salt-sensitive and salt-tolerant wheat cultivars under salinity stress. Significantly, this enhancement is attained even with the increased buildup of Na^+^ and Cl^−^ ions, suggesting that GA-mediated stress tolerance is mainly associated with enhanced physiological functioning and metabolic regulation rather than relying solely on ion exclusion strategies (Shahzad et al., 2021).

Together these results indicate that gibberellins are not only growth promoting hormones, but also important regulators of stress adaptation. GAs orchestrate the trade-off between growth, resource allocation and stress defense via interactions with DELLA proteins and other hormone signaling pathways, allowing plants to optimize survival and productivity under adverse environmental conditions.

### 2.6 Salicylic acid-mediated regulation of plant adaptation to environmental stress

The phenolic phytohormone salicylic acid (SA) is essential for controlling plant growth, development, and reactions to environmental stressors. SA is engaged in numerous physiological processes, including seed germination, blooming, photosynthesis, transpiration, thermotolerance, stomatal control, ion transport, senescence, and nodulation, in addition to its well-known function in plant immunity. By controlling the expression of stress-responsive genes and triggering several defense mechanisms, SA is a crucial signaling molecule that increases plant resistance to biotic and abiotic challenges. Additionally, SA coordinates growth-defense trade-offs and adaptive responses under stress conditions by substantial cross-talk with other phytohormones, such as auxins, cytokinins, ethylene (ET), jasmonic acid (JA), and abscisic acid (ABA) (Song et al., 2023a).

SA is most well-known for its function in plant immunity, particularly in the induction of systemic acquired resistance (SAR), a long-lasting and broad-spectrum defense response that is activated after pathogen infection. A key player in SAR is the regulatory protein NONEXPRESSOR OF PATHOGENESIS-RELATED GENES 1 (NPR1), which acts as a master regulator of SA-dependent defense signaling. Cellular redox changes after SA accumulation allow NPR1 activation and translocation into the nucleus, leading to transcriptional activation of defense-related genes including PATHOGENESIS-RELATED (PR) proteins such as PR1 and PR2. Through this signaling pathway, SA provides increased resistance to a broad spectrum of biotrophic and hemibiotrophic pathogens.

Besides its role in disease resistance, SA is also important for abiotic stress tolerance. Many studies have shown that the exogenous application of SA can improve the performance of plants under salinity stress by increasing photosynthetic efficiency, maintaining membrane integrity, regulating antioxidant defense and decreasing the accumulation of toxic ions. SA treatment has been reported to have positive effects on different crop species including mustard, bean, mung bean, barley, wheat and maize where SA enhances physiological stability and promotes stress resilience (Husen et al., 2018; Ghassemi-Golezani and Lotfi, 2015; Pirasteh-Anosheh et al., 2015; El-Nasharty et al., 2019).

Salicylic acid is also important for drought adaptation. The increase in endogenous SA levels in Phillyrea angustifolia under drought stress indicates that the accumulation of SA is a part of the plant adaptive response to water deficit (Munne-Bosch and Penuelas, 2003). SA under drought condition enhances antioxidant system activation, photosynthetic capacity maintenance, osmotic adjustment and stress responsive gene expression. Recent studies also indicate that SA improves stress-induced glucose metabolism and photosynthetic efficiency and hence helps in improving stress tolerance and productivity under adverse environmental conditions (Kumar et al., 2025).

There is a growing body of evidence suggesting that SA plays an important role in the regulation of cellular redox homeostasis. SA regulates reactive oxygen species (ROS) production and antioxidant enzyme activities, affecting stress signaling networks and defense responses. Furthermore, the extensive crosstalk between SA and other phytohormones enables plants to modulate their responses to a range of environmental obstacles. SA and JA often exhibit antagonistic regulation of defense pathways, while synergistic interactions with ABA, ET and auxin serve to integrate developmental and stress-responsive processes.

Together, these results identify SA as a major regulator of plant adaptation to environmental stress. SA is involved in the regulation of systemic acquired resistance, redox, transcriptional reprogramming, and hormonal crosstalk, coordinating different defense and acclimation mechanisms that enhance resilience in plants under biotic and abiotic stress. Nevertheless, further studies are needed to elucidate the complex molecular networks involved in SA-mediated stress adaptation.

### 2.7 Role of strigolactones in enhancing plant stress tolerance

Strigolactones (SLs) are a class of carotenoid-derived phytohormones that have important regulatory roles in plant development and environmental adaptation. They were first characterized as signaling molecules secreted by plant roots that induce the germination of parasitic plants such as Striga, Orobanche and Phelipanche species (Cardoso et al., 2011). Subsequent research revealed that strigolactones also serve as endogenous hormones regulating numerous developmental processes, including shoot branching, root architecture, secondary growth, and reproductive development. The structural diversity of strigolactones is considerable, with different plant species producing distinct mixtures and concentrations of SL molecules. This diversity reflects their broad functional roles in plant physiology and ecological interactions. Under nutrient-limiting conditions, particularly phosphorus and nitrogen deficiency, strigolactone biosynthesis is strongly induced. Elevated SL levels suppress shoot branching while promoting modifications in root system architecture, thereby enhancing nutrient acquisition and improving resource-use efficiency (Qi et al., 2024; El-Beltagi et al., 2026).

The perception and signaling of strigolactones are mediated through a well-characterized pathway involving the α/β-hydrolase receptor DWARF14 (D14) and the F-box protein MORE AXILLARY GROWTH 2 (MAX2). Upon strigolactone perception, D14 interacts with MAX2, leading to the degradation of downstream repressors and activation of SL-responsive developmental and stress-related pathways. This signaling module enables plants to integrate environmental signals with growth regulation and adaptive responses. Increasing evidence indicates that strigolactones play important roles in plant adaptation to abiotic stresses, including drought, salinity, nutrient deficiency, and temperature extremes. Under water-limited conditions, SLs contribute to stress tolerance by modulating root system architecture, enhancing water acquisition, regulating stomatal behavior, and interacting with ABA-dependent signaling pathways. Through these mechanisms, strigolactones help optimize plant growth while improving survival under adverse environmental conditions.

Strigolactones also participate in extensive hormonal crosstalk with auxins, cytokinins, ABA, and ethylene. Particularly notable is the interaction between auxin and SL signaling, which coordinates shoot branching and root development in response to environmental constraints. Such hormonal interactions enable plants to balance growth and stress adaptation according to prevailing environmental conditions.

Beyond their developmental and abiotic stress-related functions, strigolactones are increasingly recognized as important mediators of plant–microbe interactions. SLs released into the rhizosphere stimulate hyphal branching of arbuscular mycorrhizal fungi, thereby promoting beneficial symbiotic associations that enhance nutrient acquisition and stress resilience. In legumes, strigolactones also contribute to rhizobial symbiosis and nodulation processes, further supporting plant nutrition and stress adaptation (El-Beltagi et al., 2025).

From an agricultural perspective, strigolactones represent promising targets for crop improvement. Their ability to regulate plant architecture, enhance tolerance to environmental stresses, and facilitate beneficial microbial associations offers opportunities for developing climate-resilient crops. Furthermore, the capacity of synthetic strigolactone analogs to induce suicidal germination of parasitic weed seeds has attracted considerable attention as a sustainable strategy for controlling Striga and related parasitic weeds in agricultural systems.

Overall, strigolactones function as multifunctional signaling molecules that integrate plant development, environmental sensing, and stress adaptation. Their emerging roles in hormonal crosstalk, rhizosphere communication, and stress resilience highlight their importance as key regulators of plant performance under changing environmental conditions.

## 3 Phytohormonal crosstalk in plant biotic stress responses

Phytohormonal signaling networks play a central role in regulating plant immune responses by integrating growth, development, and defense pathways. Rather than acting independently, plant hormones interact through complex antagonistic and synergistic relationships that enable plants to fine-tune their responses against diverse biotic challenges. These hormonal interactions determine the balance between growth maintenance and activation of defense mechanisms during pathogen attack and herbivore infestation.

Auxins are primarily recognized as key regulators of plant growth and development; however, increasing evidence indicates that auxin signaling is closely associated with plant susceptibility to pathogens. Effective resistance against several biotic stresses frequently involves suppression or reprogramming of auxin pathways. During systemic acquired resistance (SAR), auxin-responsive gene expression is commonly reduced, indicating an antagonistic relationship between auxin and immune signaling. For instance, treatment of Arabidopsis with the salicylic acid (SA) analog benzothiadiazole S-methyl ester (BTH) results in repression of auxin-responsive genes (Oussou et al., 2026). Furthermore, SA signaling suppresses the expression of auxin receptors, including Transport Inhibitor Response 1 (TIR1) and Auxin F-Box (AFB) proteins, while promoting the accumulation of AUX/IAA transcriptional repressors. This reduces auxin-mediated transcriptional activation and redirects cellular resources toward defense responses (Bell et al., 2026).

Cytokinins (CKs) also contribute significantly to plant immune regulation through their interaction with defense-associated hormonal pathways. Several studies indicate that cytokinin signaling influences plant resistance against pathogens by modulating immune-related gene expression and interacting with SA-dependent defense mechanisms. During infection by hemibiotrophic pathogens such as Verticillium longisporum, changes in cytokinin metabolism and signaling contribute to the activation of defense responses (Nie et al., 2026). Cytokinin-induced transcription factors can enhance SA-mediated immunity, highlighting the importance of CK–SA crosstalk in disease resistance (Fàbregas et al., 2026). In rice, coordinated activation of cytokinin and SA signaling pathways contributes to enhanced resistance against rice blast disease through regulation involving OsNPR1 and WRKY45 transcription factors (Zhang et al., 2022).

Gibberellins (GAs) and jasmonates (JA) also exhibit extensive interaction during plant defense responses. GA signaling is largely controlled by DELLA proteins, which function as negative regulators of GA responses and important modulators of stress adaptation. DELLA proteins interact with JAZ proteins, the major repressors of JA signaling, thereby influencing JA-dependent defense activation (Davière and Achard, 2016). Under pathogen attack, reduced GA signaling promotes DELLA accumulation, which enhances JA-mediated defense responses by facilitating the release of JA-responsive transcription factors. In addition, JA regulates the expression of GA signaling components, including repressors such as RGL3, thereby strengthening resistance against necrotrophic pathogens. This regulation involves competition between JA-associated transcription factors, particularly MYC2, and growth-related signaling components, allowing plants to prioritize defense over growth during infection (Li and Hou, 2017).

Brassinosteroids (BRs) are another important class of growth-promoting hormones that participate in extensive crosstalk with immune-related pathways, including SA, JA, ABA, auxin, and GA signaling. BRs can enhance resistance against pathogens through both SA-dependent and SA-independent mechanisms. However, their effects on immunity are highly context-dependent, as BR signaling may also antagonize JA-mediated defense responses in certain plant species, including rice. Such interactions demonstrate the complex role of BRs in balancing growth and defense priorities. The integration of BR and auxin signaling represents an important regulatory mechanism controlling both plant development and immunity. BR and auxin pathways are involved in the regulation of cell expansion, root architecture and defense-related transcriptional responses, through bidirectional interactions (Faizan et al., 2026). Furthermore, BR signaling components regulate GA pathways by influencing GA biosynthesis and signaling repression. The BR-responsive transcription factor BZR1 interacts with DELLA proteins, creating a regulatory connection between BR and GA signaling networks that contributes to the coordination of growth and stress adaptation (Thomas et al., 2016). **Figure 3** shows the complex crosstalk of major phytohormones including SA, JA, ET, ABA, auxin, CK, BR and GA in regulating plant responses to biotic stress. These hormones interact synergistically and antagonistically to coordinate the activation of defense, resistance to pathogens and herbivores and the balance between growth and defense. These interconnected signaling networks enable plants to dynamically fine-tune immune responses according to the type and severity of biotic stress.

**Figure 3:**
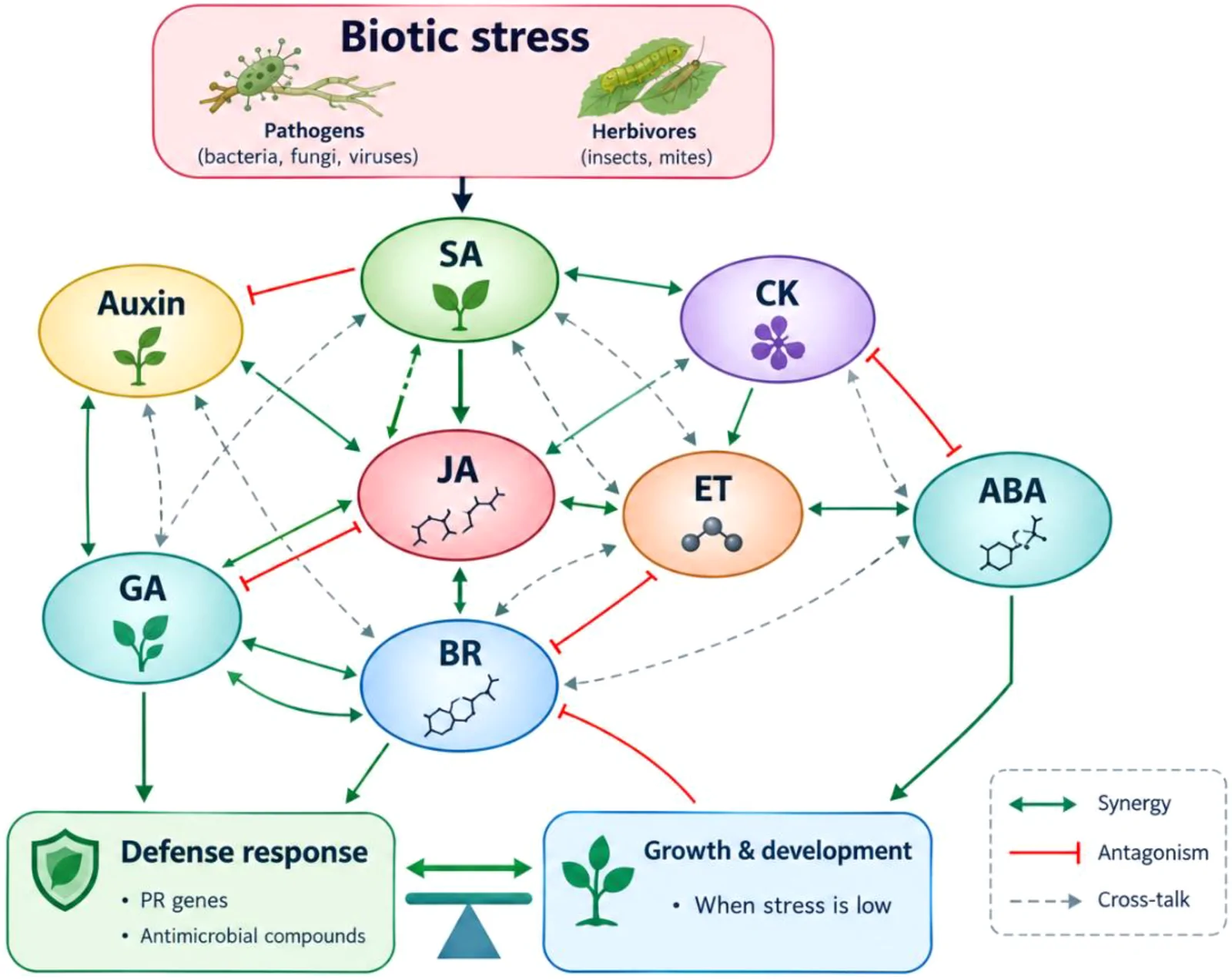
Phytohormonal crosstalk regulating plant responses to biotic stress. SA, salicylic acid; JA, jasmonic acid; ET, ethylene; ABA, abscisic acid; IAA, indole-3-acetic acid; CK, cytokinins; BR, brassinosteroids; GA, gibberellins; PR, pathogenesis-related; DELLA, DELLA growth-repressor proteins; JAZ, JASMONATE ZIM-domain proteins; SAR, systemic acquired resistance.

Overall, phytohormonal crosstalk provides plants with a flexible regulatory system that enables rapid adjustment to biotic stresses. Antagonistic interactions, such as SA-auxin and JA-GA relationships, facilitate the reallocation of resources from growth to defense. Synergistic interactions among CK, SA, BR and other hormones, increase immune responses. Understanding these complex hormonal networks provides valuable insights for improving crop resistance and developing stress-resilient agricultural systems.

Beneficial microbes establish intricate interactions with plants that can modulate phytohormonal signaling and enhance resistance to biotic stresses. Plant growth-promoting rhizobacteria (PGPR), endophytes, and mycorrhizal fungi can modulate biosynthesis, accumulation, and signaling of SA, JA, ET, auxin, cytokinins, and thus prime plants for stronger defense system (Pieterse et al., 2014). PGPR can induce systemic resistance (ISR) through microbial signals and metabolites, such as volatile organic compounds, siderophores and other elicitors, which regulate hormone-associated defense pathways and enhance plant resistance to pathogens and insect herbivores (Pieterse et al., 2014; Zhang et al., 2022). Particularly, PGPR-mediated ISR is closely related to JA- and ET-dependent signaling, but interactions with SA signaling enable plants to modulate their defense responses based on the invading organism and environmental conditions (Pieterse et al., 2014; Zhang et al., 2022).

Endophytic bacteria and fungi can also induce plant defense responses by colonizing internal plant tissues and affecting hormone production, secondary metabolism and defense gene expression. Endophyte-mediated resistance involves complex interactions between the SA, JA and ET pathways allowing plants to coordinate local and systemic defense responses without compromising growth (Mohammed et al., 2026; Talukdar et al., 2026). Mycorrhizal associations, particularly those established by arbuscular mycorrhizal fungi, also influence plant hormone networks and can induce mycorrhiza-induced resistance where colonization of the root system primes defense responses in roots and distal tissues.

Importantly, plant-microbe-hormone interactions do not involve only single hormone pathways, but are characterized by extensive crosstalk with reactive oxygen species (ROS), Ca^2+^ signaling, mitogen-activated protein kinase (MAPK) cascades, and transcriptional regulators. Thus, beneficial microbes can be a signaling partner, modulating the plant immune state and improving the defense readiness, while minimizing the metabolic costs for constitutive defense activation (Zhang et al., 2022). Microbe-triggered ROS generation and Ca^2+^ signaling can activate downstream MAPK cascades and defense-related transcription factors, providing an important link between microbial perception and hormone-dependent immune responses. Thus, PGPR, endophytes and mycorrhizae should be viewed as integral components of the plant-microbe-hormone signaling network contributing to plant coordination of growth and defense and enhancing resistance to pathogens and herbivores (Pieterse et al., 2014; Begum et al., 2019; Talukdar et al., 2026).

## 4 Integration of hormonal crosstalk in abiotic stress regulation

The adaptation of plants to abiotic stress is mediated by complex hormonal networks where multiple phytohormones interact to control physiological, molecular and developmental responses (**Figure 4**). Among these hormones, abscisic acid (ABA) is thought to be one of the earliest and most important stress-responsive signals. Under conditions such as drought, salinity and osmotic stress, ABA rapidly accumulates and participates in the regulation of water balance, stomatal movement and stress-responsive gene expression. The importance of ABA signaling in stomatal control is clear in mutants with deficiencies in ABA biosynthesis, perception or signal transduction that often fail to properly regulate stomatal aperture and show severe wilting phenotypes even in the absence of stress in plants such as Arabidopsis and tomato (Korwin Krukowski et al., 2020).

**Figure 4:**
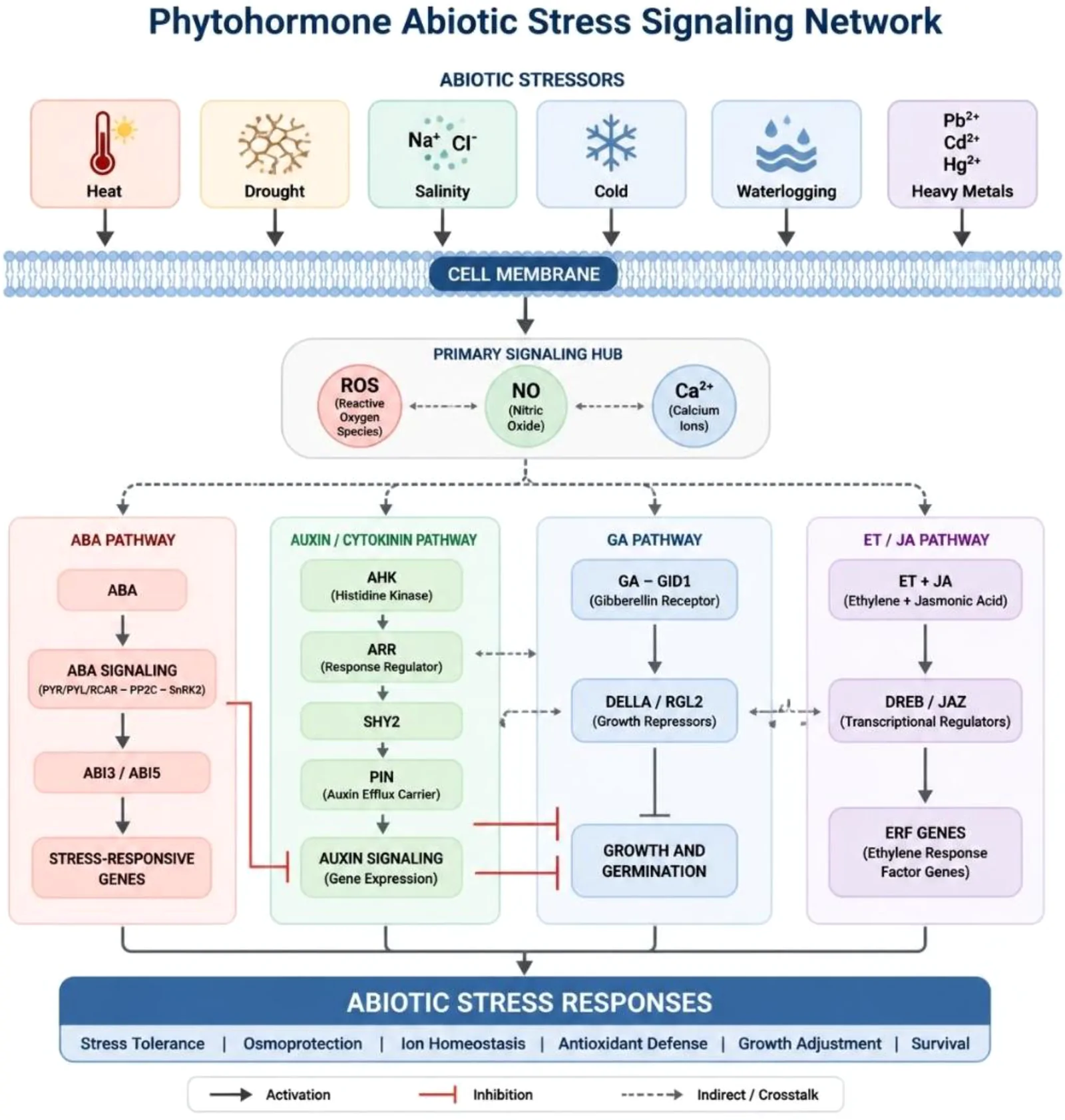
Crosstalk of various hormones to upregulate and downregulate different mechanisms to ameliorate abiotic stresses.

While ABA is a major regulator of stomatal closure, more and more evidence suggests that stomatal behavior is governed by a complex hormonal signaling network, rather than solely by ABA. Other signaling molecules, including brassinosteroids (BRs), salicylic acid (SA), jasmonic acid (JA), nitric oxide (NO), and reactive oxygen species (ROS), interact with ABA pathways to modulate guard cell responses according to environmental conditions (Wei et al., 2021a). These interactions enable the plants to maximize water conservation strategies, while keeping growth and metabolic activity.

Nitric oxide (NO) is an important signaling intermediate in ABA-mediated stress responses. ABA-induced stomatal closure is usually accompanied by NO accumulation in guard cells, indicating that NO functions downstream of ABA or as a parallel signaling component. NO promotes ABA-dependent responses and participates in stomatal regulation and general stress adaptation processes by interacting with calcium signaling, ROS metabolism, and protein modifications (Xu et al., 2024).

Another important regulator of tolerance to abiotic stresses is jasmonic acid (JA). JA accumulation is up-regulated in response to several environmental factors including drought, mechanical damage, herbivore attack and necrotrophic pathogen infection. JA extensively interacts with ABA signaling pathways to regulate stress-responsive gene expression and physiological adaptation in drought. JA and ABA affect common processes such as growth inhibition, control of seed germination, induction of senescence and activation of defense-related metabolic pathways (Jang et al., 2020).

The crosstalk between ABA and JA is of particular significance as both hormones modulate common transcriptional networks. For example, ABA and JA jointly regulate the expression of storage related genes, such as, the expression of napin and oleosin genes. Moreover, JA-induced stress responses seem to be partially dependent on increased endogenous ABA accumulation, indicating that ABA acts as a mediator that integrates JA signaling with downstream stress adaptation pathways (Hays et al., 1999). This hormonal cross-talk enables plants to coordinate the activation of defenses and allocation of resources under water limitation.

Another important aspect of stress regulation is the crosstalk between the jasmonic acid and salicylic acid. Conventional wisdom has it that the JA and SA pathways are antagonistic, especially when combined with plant immune responses. SA-dependent resistance to biotrophic pathogens often involves suppression of JA-dependent resistance to necrotrophic pathogens and herbivores. In Arabidopsis, the accumulation of SA in response to pathogens is often accompanied by the suppression of JA signaling, allowing plants to activate the most suitable defense strategy depending on the pathogen encountered (Hou and Tsuda, 2022). However, this antagonism is highly context dependent and JA and SA can also function synergistically under certain abiotic stresses. Both hormones are involved in thermotolerance by enhancing antioxidant capacity and heat stress-responsive mechanisms (Zhang et al., 2025). Heat tolerance is highly linked to the induction of heat shock proteins (HSPs) which act as molecular chaperones and maintain protein stability, prevent aggregation and mediate protein refolding under high temperature conditions. SA and JA are involved in this process by modulating stress-responsive transcriptional networks and cellular homeostasis (Khan and Shahwar, 2020).

Hormonal interactions also control developmental adjustments under water stress. Leaf and fruit abscission are typical responses to drought and are mostly regulated by the cross-talk between ABA and ethylene signaling pathways. Although the process is also regulated by cytokinins (CKs), ABA–ethylene crosstalk is the dominant player in the regulation of senescence and organ shedding under stress conditions (Singh and Roychoudhury, 2023). The exogenous application of benzyl adenine (BA), a synthetic cytokinin, modifies source-sink relationships, by changing the expression of genes related to ethylene, ABA, cytokinin degradation and gibberellin metabolism and affecting the allocation of nutrients between fruits and leaves. These results suggest that cytokinins regulate abscission indirectly by interacting with multiple hormonal pathways at the same time (Mangena, 2022).

Ethylene Response Factor 1 (ERF1) acts as an essential regulatory bridge connecting ethylene signaling to other stress-related hormonal pathways. The interaction of ERF1 with mitogen-activated protein kinase 1 (MAPK1) and its induction by ABA and SA under stress conditions suggest that ERF1 plays a central role in integrating hormonal signaling networks. Induction of maize ERF1 expression in response to ABA treatment also supports functional interactions of the ethylene and ABA pathways during stress adaptation (Li et al., 2022b; Shi et al., 2016).

Auxin also mediates abiotic stress through complex interactions with ABA and ethylene. Auxin and ethylene often interact synergistically in the regulation of plant growth and development, but under stress conditions, their interactions can become antagonistic. Auxin transport from shoots to roots can induce ethylene production, which influences auxin distribution and inhibits cell elongation in the root elongation zone. This mechanism limits root growth under unfavorable environmental conditions and contributes to developmental adjustment in response to stress (Liu et al., 2017; Zou et al., 2021).

An additional significant hormonal component linking growth regulation and stress adaptation are gibberellins (GAs). GA signaling regulates seed germination, vegetative growth, flowering and developmental transitions. The central players in GA signaling are the GA receptor GID1, DELLAs and SCF-type E3 ubiquitin ligase complexes with F-box proteins such as SLEEPY1 (SLY1) and SNEEZY (SNZ). In the presence of bioactive GA, the hormone binds to GID1 receptor, forming a GA–GID1 complex that interacts with DELLA proteins. Such interaction facilitates the recruitment of the SCF-SLY1/GID2 complex, leading to the ubiquitination and subsequent degradation of DELLA proteins via the 26S proteasome pathway. DELLA proteins are growth repressors and their degradation activates GA-dependent growth processes (Gao et al., 2011). However, under stress conditions, decreased GA activity results in the accumulation of DELLA proteins, allowing plants to inhibit growth and divert resources to survival and stress tolerance.

To summarize, a phytohormonal crosstalk provides a highly flexible regulatory framework for plants to cope with environmental stress. Hormones such as ABA, JA, SA, ET, auxins, cytokinins, BRs and GAs do not operate through isolated pathways but extensively cross-talk to coordinate water conservation, defense activation, metabolic adjustment and growth regulation. These interconnected networks need to be understood to develop crops with improved resilience under changing environmental conditions.

## 5 Molecular signaling networks in phytohormonal stress responses

The responses of plants to abiotic and biotic stresses are regulated by interconnected molecular signaling networks that convert environmental cues into transcriptional and physiological responses. Stress perception quickly activates secondary messengers, particularly Ca²^+^ and reactive oxygen species (ROS), which function as signaling molecules and have extensive cross-talk with phytohormonal pathways. Calcium sensors, such as calmodulins, calcium-dependent protein kinases (CDPKs), and CBL–CIPK complexes, interpret stress-induced Ca²^+^ signatures and control ion transport, transcription factors, antioxidant systems, and hormone-responsive genes (Ravi et al., 2023; Naz et al., 2024). Meanwhile, ROS generated under stress act as signaling molecules and redox regulators, interacting with ABA, JA, SA, and ethylene pathways to coordinate defense and acclimation responses (Li et al., 2022; Samanta et al., 2024).

Stress perception is also associated with downstream gene regulation by protein kinase cascades, particularly mitogen-activated protein kinase (MAPK) pathways. These pathways integrate Ca^2+^, ROS and phytohormonal signals to modulate transcription factors and stress-responsive genes, thus coordinating processes such as stomatal regulation, antioxidant defense, osmotic adjustment and pathogen resistance. Importantly, hormone signaling is not linear but contains extensive synergistic and antagonistic interactions. For example, ABA-ROS interactions are key to drought-induced stomatal closure, while JA, SA and ethylene signaling are strongly interconnected during immune responses (Li et al., 2022; Wang et al., 2025). Thus, Ca^2+^, ROS, cascades of protein kinases and phytohormonal pathways form an integrated signaling network rather than independent pathways that provide plants with the molecular plasticity needed to balance growth, defense and stress adaptation (Waadt et al., 2022; Ravi et al., 2023).

## 6 Epigenetic regulation and stress memory

Phytohormonal signaling represents a central regulatory layer through which plants sense and respond to abiotic and biotic challenges, but an increasing body of evidence suggests that epigenetic mechanisms also contribute to these responses (Lämke and Bäurle, 2017; Bäurle and Trindade, 2020). DNA methylation, histone modifications, chromatin remodeling, and non-coding RNAs (ncRNAs) constitute a regulatory layer that affects the accessibility and transcriptional activity of stress-responsive genes, and contribute to the establishment of stress memory. Unlike genetic alterations, epigenetic modifications are potentially dynamic and reversible such that plants can rapidly modulate gene expression in response to environmental conditions while still maintaining a “memory” of prior stress experience (Lämke and Bäurle, 2017).

DNA methylation is a major component of plant stress response, primarily in CG, CHG and CHH sequence contexts. Environmental stresses like drought, salinity, temperature extremes and pathogen infection can alter DNA methylation patterns in some genomic regions and thus affect the expression of stress-responsive genes (Kim, 2021). DNA methyltransferases and RNA-directed DNA methylation (Rd.) pathways may mediate stress-induced changes in DNA methylation, linking environmental signals to transcriptional regulation. Many stress-induced methylation changes are transient and revert to baseline levels after recovery but some changes can be persistent and contribute to stress priming or memory (Lämke and Bäurle, 2017). Therefore, DNA methylation could be an epigenetic interface that connects environmental sensing to long-term regulation of plant stress responses.

Another important mechanism in regulation of stress-responsive genes is the modification of histones and remodeling of chromatin. Generally, histone acetylation is involved in enhanced chromatin accessibility and transcriptional activation, while histone deacetylation can trigger transcriptional repression (Bäurle and Trindade, 2020; Kim, 2021). Similarly, histone methylation may activate or repress transcription depending on the residue modified. For example, H3K4me3 is generally associated with transcriptionally active chromatin while H3K27me3 is often associated with gene repression. Changes in these histone marks in response to stress can facilitate the rapid activation or repression of genes involved in antioxidant defense, osmotic adjustment, defense against pathogens and hormone signaling (Bäurle and Trindade, 2020). Chromatin remodeling complexes are also involved in stress memory by controlling nucleosome positioning and maintaining transcriptionally competent states at previously activated stress-responsive loci (Brzezinka et al., 2016; Bäurle and Trindade, 2020).

Non-coding RNAs (ncRNAs) such as microRNAs (miRNAs), small interfering RNAs (siRNAs), long non-coding RNAs (lncRNAs) and other regulatory RNAs add another layer of regulation linking stress signaling to epigenetic and post-transcriptional regulation. MiRNAs and siRNAs can regulate transcripts encoding transcription factors and components of hormone signaling pathways, and siRNAs can be involved in RdDM-mediated DNA methylation (Abdulraheem et al., 2024). LncRNAs may interact with transcription factors, chromatin-modifying proteins, or target RNAs to modulate gene expression in response to stress. Hence, ncRNAs could act as molecular bridges in phytohormonal signaling, transcriptional control and chromatin remodeling (Sintaha, 2025).

In addition, these epigenetic pathways also play a role in stress memory, in which previous stress experiences result in a faster or stronger response to a future stress. Somatic stress memory can be described by stable alterations in histone modifications, nucleosome occupancy, DNA methylation and stress-responsive transcription (Bäurle and Trindade, 2020). Hormones such as ABA, jasmonic acid (JA), salicylic acid (SA) and ethylene are strongly linked to this process and can work with chromatin based pathways to develop or maintain primed states. ABA signaling, for example, can affect chromatin regulators involved in drought and heat responses, and SA- and JA-dependent pathways can modulate the transcriptional status of genes (Siddique et al., 2024).

Intergenerational and transgenerational consequences have been related to stress-induced epigenetic modifications but these must not be confused with somatic stress memory. Stress-induced epigenetic changes are often reversible and only few are expected to survive meiosis and generations. However, the ability of plants to integrate hormone signals with chromatin states and ncRNA-based regulation provides a fundamental molecular basis for stress priming and adaptation (Lämke and Bäurle, 2017). Thus, the interaction between phytohormonal crosstalk and epigenetic control adds another layer of regulation to plant defense, which may lead to new avenues for the production of crops with enhanced and long-lasting tolerance to recurrent abiotic and biotic challenges.

## 7 Conclusion and future perspectives

Phytohormones are key regulators that link environmental signals to developmental programs and enable plants to cope with biotic and abiotic stresses. ABA plays a key role in adaptation to abiotic stress through regulation of stomatal closure, water-use efficiency, osmotic adjustment, antioxidant defense and stress-responsive gene expression. SA and JA are key regulators of plant immunity. Their crosstalk helps to balance the defense activation, growth and resource allocation. Auxins and CKs coordinate stress-induced developmental plasticity by regulating root architecture, cell division, senescence, and recovery, while ET is involved in the coordination of defense, senescence, growth inhibition, and acclimation. GAs, BRs and SLs also play a role in the growth and survival of plants in challenging environments. There is increasing evidence that these hormonal pathways are integrated with ROS, Ca ^2+^ signaling, secondary metabolites, transcription factors, epigenetic modifications and RNA-mediated regulation, creating highly dynamic regulatory networks that can generate stress memory and priming. Nevertheless, there are crucial knowledge gaps in how phytohormonal networks perceive, integrate and priorities multiple environmental signals. Most mechanistic studies have been conducted on individual stresses under controlled conditions, but plants in natural and agricultural environments are often subjected to combined or sequential stresses. Future research should therefore be paid to the spatial regulation of hormone biosynthesis, transport, perception and signaling between different tissues and cell types during stress and recovery. Advanced multi-omics approaches, including transcriptomics, proteomics, metabolomics, epigenomics, spatial transcriptomics and single-cell sequencing, offer powerful opportunities to resolve cell-specific hormonal responses and to identify regulatory hubs controlling stress adaptation. Crucially, future studies should move beyond descriptive omics datasets to experimentally validated predictive models that link molecular changes with physiological performance and yield.

Crosstalk between phytohormonal networks and the plant microbiome is another promising avenue for sustainable crop improvement. Plant-growth-promoting rhizobacteria (PGPR), endophytes and mycorrhizal fungi are beneficial microorganisms which can affect hormone biosynthesis and signaling, enhance nutrient acquisition, root development, antioxidant capacity and stress tolerance (Aloo et al., 2023). Future research should increasingly investigate these plant–microbe–hormone interactions under realistic field conditions, and explore whether microbial consortia can be rationally designed to modulate specific hormonal pathways.

Artificial intelligence (AI) and machine learning (ML) provide an emerging opportunity to propel this transition from descriptive to predictive plant stress biology. AI-based methods can combine large datasets from multi-omics platforms, environmental sensors, imaging systems and high-throughput phenotyping to identify complex relationships between hormonal signatures, environmental conditions and stress-related phenotypes (Paul et al., 2025). Therefore, future efforts should combine AI predictions with mechanistic experiments to distinguish biologically meaningful regulatory relationships from statistical associations. Another important frontier for the translation of molecular knowledge into practical crop management is the integration of phytohormonal signaling with precision agriculture. Linking measurements obtained by soil and plant sensors to physiological and hormonal indicators would enable site-specific management of irrigation, fertilization, and stress based on the actual physiological needs of crops. Such integration could result in major gains in water and nutrient-use efficiency and reductions in superfluous agricultural inputs. A major future direction is the development of integrated digital phenotyping and hormonal-stress signatures, where molecular and physiological indicators are combined with real-time environmental and remote-sensing data. For example, changes in ABA, SA, JA, ET, ROS, Ca^2+^ signaling, stomatal behavior or stress-responsive gene expression could be integrated with canopy temperature, spectral indices, soil moisture and microclimatic measurements. AI-driven models could then reveal early warning signatures of stress and support timely management interventions prior to substantial yield losses. Such approaches may help to bridge the gap between molecular plant biology, crop physiology and field-scale precision agriculture (Koh et al., 2024).

Integration of these technologies with multi-omics and AI-assisted target discovery may allow identification and validation of key regulatory genes and signaling nodes associated with stress resilience. However, improving stress tolerance should be considered in conjunction with yield, quality, and resource-use efficiency. Thus future agricultural innovation should move away from increasing tolerance of individual stresses to integrated and predictive resilience to multiple environmental constraints. A major challenge and opportunity will be to integrate phytohormonal signaling, multi-omics, microbiome engineering, AI, precision agriculture and advanced breeding within a single framework.
